# Temporal clustering of surgical activities in robot-assisted surgery

**DOI:** 10.1007/s11548-017-1600-y

**Published:** 2017-05-05

**Authors:** Aneeq Zia, Chi Zhang, Xiaobin Xiong, Anthony M. Jarc

**Affiliations:** 1 College of Computing, Georgia Institute of Technology, North Ave NW, Atlanta, GA 30332 USA; 2 Electrical Engineering and Computer Science, University of Tennessee, 1520 Middle Dr, Knoxville, TN 37996 USA; 3 Robotics, Georgia Institute of Technology, North Ave NW, Atlanta, GA 30332 USA; 4 Medical Research, Intuitive Surgical, Inc., 5655 Spalding Drive, Norcross, GA 30092 USA

**Keywords:** Robot-assisted surgery, Segmentation, Clustering, Performance evaluation

## Abstract

**Purpose:**

Most evaluations of surgical workflow or surgeon skill use simple, descriptive statistics (e.g., time) across whole procedures, thereby deemphasizing critical steps and potentially obscuring critical inefficiencies or skill deficiencies. In this work, we examine off-line, temporal clustering methods that chunk training procedures into clinically relevant surgical tasks or steps during robot-assisted surgery.

**Methods:**

We collected system kinematics and events data from nine surgeons performing five different surgical tasks on a porcine model using the da Vinci Si surgical system. The five tasks were treated as one ‘*pseudo-procedure*.’ We compared four different temporal clustering algorithms—hierarchical aligned cluster analysis (HACA), aligned cluster analysis (ACA), spectral clustering (SC), and Gaussian mixture model (GMM)—using multiple feature sets.

**Results:**

HACA outperformed the other methods reaching an average segmentation accuracy of $88.0\%$ when using all system kinematics and events data as features. SC and ACA reached moderate performance with $84.1\%$ and $82.9\%$ average segmentation accuracy, respectively. GMM consistently performed poorest across algorithms.

**Conclusions:**

Unsupervised temporal segmentation of surgical procedures into clinically relevant steps achieves good accuracy using just system data. Such methods will enable surgeons to receive directed feedback on individual surgical tasks rather than whole procedures in order to improve workflow, assessment, and training.

## Introduction

Over the course of entire procedures, surgeons perform certain tasks that are more critical than others. For example, during a prostatectomy, surgeons must finely coordinate their tools to carefully avoid damaging nerves during the dissection of the neurovascular bundles, whereas mobilizing the colon and dropping the bladder do not involve similar risks. Despite these apparent differences across steps, most evaluations of surgical workflow or surgeon skill at population scales use simple, descriptive statistics (e.g., time) across whole procedures, thereby deemphasizing critical steps and potentially obscuring critical inefficiencies or skill deficiencies. If we could develop tools and algorithms to automatically recognize clinically relevant surgical tasks within procedures, we might be able to improve surgical workflow [1], skill assessment [2, 3], surgeon training, and, ultimately, patient safety by providing task-specific performance measures.

**Fig. 1:**
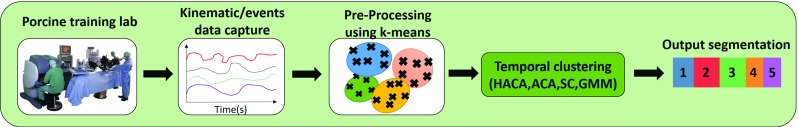
Flow diagram of the proposed model for unsupervised surgical phase segmentation

Multiple approaches to recognize surgical tasks have been proposed previously for laparoscopic [1, 4–6], ENT [7], cataract [8], and robot-assisted surgery (RAS) [9–14]. Some of these methods focus on low-level trajectories to build a surgical grammar that could be used to identify higher-level tasks or to develop surgical automation routines. Although extensive manual annotation of the datasets was required to train models to recognize the low-level features, several groups have proposed unsupervised methods to identify similar low-level trajectories with strong alignment to human labels [13, 14].

More recently, researchers have started to develop vision-based methods to recognize higher-level tasks, such as clinical steps of a procedure, from laparoscopic videos [4, 15, 16]. In this way, video clips are the only input to these algorithms. The majority of approaches borrow from recent successes in deep learning using convolutional neural networks (CNNs) and recurrent neural networks (RNNs). The results have been impressive, resulting in greater than $80\%$ accuracy on certain datasets [4, 16]. Furthermore, these models often have the added advantage of providing real-time state estimation.

Despite the recent successes of video-based methods, there remain compelling reasons why one would (a) want to use smaller data streams than video and (b) utilize off-line methods without real-time capability. Small data streams enable feasible storage of data across many procedures, streaming of data over network connections without large bandwidth or disruption, and smaller compute resources for training the models. Using non-video data strongly parallels research directions in activity recognition where wearables with simple accelerometer signals might be used. Additionally, off-line methods can utilize data from entire procedures for phase recognition and remain useful for post-operative feedback, review, and documentation by surgeons. For these reasons, we believe system data from robotic surgical systems offer a scalable, practical approach to surgical segmentation and skill estimation.

Here, we examine temporal clustering methods to perform off-line surgical task recognition using only non-video data from RAS systems. In particular, we apply models developed for human activity recognition [17, 18]. We explore our models using data from clinically relevant tasks performed on porcine models in a training environment. Furthermore, novice RAS surgeons across multiple specialties performed all tasks which increased the variability in system use and strategy and, in turn, increases the generalizability of our models.

In the end, the main contributions of our work are as follows: (1) we propose a novel approach to use temporal clustering algorithms to recognize high-level surgical phases using the relatively lightweight data from RAS surgical platforms, and (2) we develop our models on realistic porcine tasks with a large amount of variability in task performance by surgeons with varying backgrounds.

## Methodology

In this section, we describe our proposed approach for unsupervised segmentation of RAS procedures. Figure 1 shows a flow diagram of our method. We collect kinematic and events data from the da Vinci Si^®^ surgical system (Intuitive Surgical, Inc., Sunnyvale, CA), while surgeons of varying expertise perform exercises on a porcine model (additional details on dataset are given in Section “Experimental evaluation”). The events data stream is used directly, whereas the kinematic time series is preprocessed before implementing different segmentation algorithms. In this paper, we propose to use aligned cluster analysis (ACA) [17] and hierarchical aligned cluster analysis (HACA) [18] for our surgical procedure segmentation since both these algorithms have proven to work well for human activity segmentation. For comparison, we also employ two additional temporal clustering algorithms: Gaussian mixture models (GMM) and spectral clustering (SC). Descriptions of the clustering algorithms are given below.

### Spectral clustering

Spectral clustering (SC) is a graph-based clustering algorithm which has been widely used for image segmentation in the computer vision community. It has also been used for time series segmentation in various biomedical applications [19]. For a given time series $T \in \mathfrak {R}^{d \times N}$, SC divides the temporal data depending on a similarity measure $s_{ij}$ between pairs of data frames $t_i$ and $t_j$. The data are represented as a similarity graph $G = (V,E)$, where *V* is the vertex set and *E* is the edge set. Each vertex of the graph $v_i$ is represented by a data frame $t_i$, and any two vertices are connected via a Gaussian similarity measure $s_{ij} = \hbox {exp}(-\frac{||t_i - t_j||^2}{2\sigma ^2})$. Once the graph *G* is constructed, the problem of clustering becomes a graph partitioning task. Therefore, in order to cluster different surgical procedures in our dataset, we partition the graph constructed so that the edges between different groups have small weights and the edges within a group have large weights.

### Gaussian mixture models

Gaussian mixture model (GMM) is a popular clustering algorithm and has been extensively used for various applications. The use of GMM for time series segmentation was originally proposed in [20]. We use a GMM to model our time series $T \in \mathfrak {R}^{d \times N}$ and segment the series whenever two consecutive frames belong to different Gaussian distributions. This is done since data frames from different surgical tasks, or activities in general, would potentially form distinct clusters which can be modeled using Gaussian distributions. We use the Expectation Maximization (EM) algorithm to estimate the parameters of each of the Gaussians in the GMM.

**Fig. 2:**
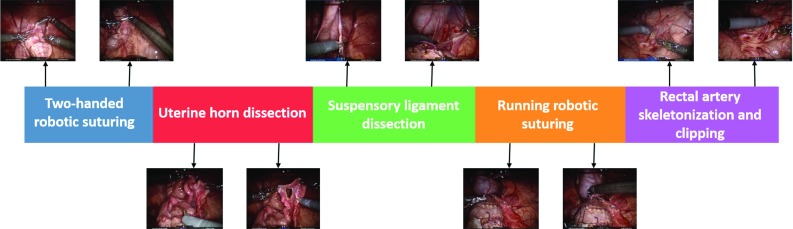
‘*pseudo-procedure*’ with sample frames for each of the five surgical tasks in the dataset

### Aligned cluster analysis and hierarchical aligned cluster analysis

Given a time series $T \in \mathfrak {R}^{d \times N}$, aligned cluster analysis (ACA) and hierarchical aligned cluster analysis (HACA) algorithms are formulated to decompose *T* into *M* different segments with each segment corresponding to one of the *K* clusters. Each segment $Q_m$ consists of frames of data from position $t_m$ till $t_{m+1}-1$, where $t_m$ and $t_{m+1}-1$ represent the first and the last index of the *m*th segment. In order to control the temporal regularity, the length of each segment $Q_m$ is constrained to the range $l_i \in [l_\mathrm{min},l_\mathrm{max}]$. A binary indicator matrix $G \in \mathfrak {R}^{K \times M}$ is generated where $g_{k,m}=1$ if the *m*th segment belongs to the *k*th cluster, otherwise $g_{k,m}=0$. The objective function for the segmentation problem is formulated as an extension to previous work on kernel *k*-means and is given by:

1 $$\begin{aligned} J_\mathrm{ACA}(G,s) = \sum \limits _{k=1}^{K} \sum \limits _{m=1}^{M} g_{k,m}D^{2}_{\psi }(Q_m,z_k) \end{aligned}$$

where the distance function $D^{2}_{\psi }(Q_m,z_k) = ||\psi (T_{[t_i,t_i+1]})-z_k||^2$, $Q_m$ represents a segment, *s* is a vector containing the start and end of each segment and $z_k$ is the geometric centroid of the *k*-th class. Just like kernel *k*-means, the distance between a segment and a class centroid is defined using a nonlinear mapping $\psi (.)$, given by

2 $$\begin{aligned} D^{2}_{\psi }(Q_m,z_k)= & {} \tau _{mm} - \frac{2}{M_k} \sum \limits _{j=1}^{M}g_{kj}\tau _{mj}\nonumber \\&+ \frac{1}{M_k^2}\sum \limits _{j_1,j_2=1}^{M}g_{kj_1}g_{kj_2}\tau _{j_1j_2} \end{aligned}$$

where $M_k$ denotes the number of segments belonging to class *k*. The dynamic kernel function $\tau$ is defined as $\tau _{ij}=\psi (Q_i)^T\psi (Q_j)$. In matrix form, the objective function for ACA can be written as

3 $$\begin{aligned} J_\mathrm{ACA}(G,H) = tr((I_m-G^T(GG^T)^{-1}G)H(F \circ W)H^T)\nonumber \\ \end{aligned}$$

where *W* is the normalized correspondence matrix, *H* is the segment indicator matrix and *F* is the frame kernel matrix, as defined in [18]. For our analysis, frame kernel matrix is of particular interest since the preprocessing parameters depend on it. Given a time series $T \in \mathfrak {R}^{d \times N}$, the frame kernel matrix $F \in \mathfrak {R}^{N \times N}$ is given by

4 $$\begin{aligned} F = \phi (T)^T\phi (T) \end{aligned}$$

Each element of the matrix $f_{ij}$ represents the similarity between the corresponding frames, $t_i$ and $t_j$, using a kernel function. We use a Gaussian kernel function for evaluating the frame kernel matrix giving $f_{ij} = \hbox {exp}(-\frac{||t_i - t_j||^2}{2\sigma ^2})$. Once the energy function $J_\mathrm{ACA}$ is formulated, a dynamic programming-based approach is used to solve for the optimal $G \in \mathfrak {R}^{K \times M}$ and $s \in \mathfrak {R}^{M+1}$ [18].

For hierarchical aligned cluster analysis (HACA), the same steps as described above for ACA are performed in a hierarchy at different temporal scales reducing the computational complexity; HACA first searches in a smaller temporal scale and propagates the result to larger temporal scales. Temporal scales over here refers to the number of segments the time series is randomly segmented into initially; a larger scale would mean less number of segments. We use a two-level HACA; the maximum segment length is restricted to $l^{(1)}_\mathrm{max}$ and $l^{(2)}_\mathrm{max}$ for the first and second levels in the hierarchy, respectively, where $l^{(1)}_\mathrm{max}<l^{(2)}_\mathrm{max}$. Please see [18] for a more detailed description of ACA and HACA.

## Experimental evaluation

### Dataset

Data were collected from nine RAS surgeons operating the da Vinci Si surgical system. Informed consent was obtained from all individual surgeons included in the study (Western IRB, Inc. Puyallup, WA). None of the surgeons had performed previous RAS procedures, but they all had prior laparoscopic and/or open experience. Five of the surgeons specialized in general surgery, three specialized in urology, and one specialized in gynecology. Each of the surgeons performed multiple training tasks in a single sitting on a porcine model that focused on the technical skills used during dissection, retraction, and suturing. During each exercise, instrument kinematics, system events, and endoscope video were recorded and synchronized. System data were recorded at 50 Hz, whereas endoscope video was recorded at 25 fps.

**Table 1:** Details of the five surgical tasks used in this study

Task	Name	Mean time (s)	Standard deviation time (s)
1	Two-handed robotic suturing	1329.2	733.9
2	Uterine horn dissection	2159.7	492.6
3	Suspensary ligament dissection	1999.3	1097.5
4	Running robotic suturing	617.6	126.7
5	Rectal artery skeletonization and clipping	1474.7	276.3

We selected five representative tasks for this study (see Table 1). The five tasks were treated as one ‘pseudo-procedure’ in our analysis as shown in Fig. 2. The video data were used to generate ground truth segmentations and was not added as a source of features in our models. All tasks were performed in the pelvis of the porcine model, and the setup joints (therefore, remote centers of motion) were unchanged for all tasks. The five tasks were performed on common anatomy within the pelvis thus ensuring that the segmentation algorithms are not simply using positions in the world reference frame to differentiate activities. Additional details about the instrument kinematic and system events data are given below.

#### Kinematic data

The kinematic data captured from the da Vinci Si surgical system consisted of the endpoint pose and joint angles from the hand controllers on the surgeon side console (SSC) and the instruments and camera on the patient-side cart (SI). The kinematic data stream from SSC consisted of a 56-dimensional time series, whereas SI was a 156-dimensional time series. We used individual data streams along with their different combinations in order to find the data stream most useful for segmenting different surgical tasks.

#### Events

A subset of the available system events were used in this study. The events used included camera control, master clutch for each hand controller, instrument following state for three patient-side arms, energy activation, and surgeon head in/out of the console. All events were represented as binary on/off time series. In total, the events data was an eight-dimensional time series.

**Fig. 3:**
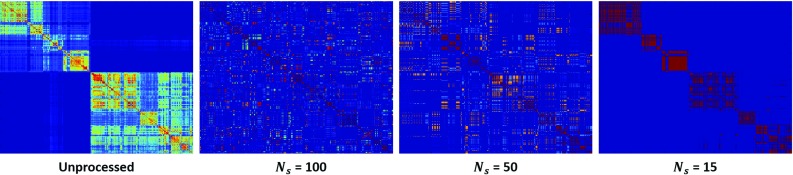
Sample frame kernel matrices for different number of symbols used in the preprocessing step. The *left* most image represents the frame kernel matrix when the time series is not reduced using k-means

### Parameter estimation

The performance of each proposed clustering algorithm depends on various parameters at each step of the pipeline. We used a subset of five randomly selected ‘pseudo-procedures’ to estimate the different parameters empirically. The details are given below.

In the preprocessing step for kinematic data, we use *k*-means clustering per trial to convert the high-dimensional time series data into symbols. The number of symbols, $N_s$, used in this step is important for the clustering performance since selecting too few symbols would fail in capturing enough information to differentiate the surgical tasks. The structure of the frame kernel matrix *F*, as described in Section “Experimental evaluation,” highly depends on the value of $N_s$. Ideally, in order to temporally segment different surgical tasks, we would want *F* to have a block structure along its diagonal. A block structure of *K* would mean a high variability in frames between different surgical tasks, and a low variability within each task. In [18], the authors selected the number of symbols (or clusters) based on characteristics of the synthetic or real data and made sure the chosen number of symbols was greater than the number of activities to be recognized. Here, we performed a coarse parameter search for the number symbols by running our clustering algorithms for a range of $N_s \in [10,15,20,50,100,150,200]$ and evaluated the clustering accuracies (using Eq. 6) for the selected subset of ‘pseudo-procedures.’ The value of $N_s$ corresponding to the highest average clustering accuracy (over the subset of ‘pseudo-procedures’) was then selected. We found that having a smaller value of $N_s$ gave better performance, with the highest average clustering accuracy being achieved with $N_s=15$. Figure 3 shows example frame kernel matrices for the same time series data but with different value of $N_s$. One can see that using fewer symbols results in a more block-like structure in the frame kernel matrix. We used 15 symbols to represent our multi-dimensional time series before employing the temporal clustering algorithms.

For the proposed clustering algorithms of ACA and HACA, the main parameter to fine-tune is the maximum segment length $l_\mathrm{max}$. ACA and HACA divide the time series into many small segments which are then assigned to different clusters. The lengths of these segments need to be selected in a way that maximizes segmentation performance. Keeping $l_\mathrm{max}$ too big would result in misclassifications at the boundaries between different tasks, whereas a smaller $l_\mathrm{max}$ would not allow for the algorithm to capture the temporal structure of the data required for segmentation. In [18], the length constraints were again chosen based on characteristics of the datasets, similar to the number of clusters, $N_s$, without formal optimization. Therefore, we empirically selected the maximum segment lengths as $l_\mathrm{max}=30$ for ACA, and $l_\mathrm{max}^1=20$ and $l_\mathrm{max}^2=30$ for the two levels in HACA, respectively, based on the length of our tasks (see Table 1 and recording rate).

### Evaluation metric

In order to evaluate the clustering accuracy for each algorithm, we calculated the confusion matrix between the ground truth $(G_\mathrm{true},H_\mathrm{true})$ and the segmentation output from the algorithm $(G_\mathrm{out},H_\mathrm{out})$. The confusion matrix $C \in \mathfrak {R}^{K \times K}$ is given by:

5 $$\begin{aligned} C = G_\mathrm{out}H_\mathrm{out}H_\mathrm{true}^{T}G_\mathrm{true}^{T} \end{aligned}$$

where each element $c_{c_ic_j}$ represents the number of frames that are in cluster segment $c_i$ and are shared by cluster segment $c_j$ in ground truth. Once the confusion matrix is calculated, we use the Hungarian algorithm [21] to find the optimum cluster correspondence giving the clustering accuracy as:

6 $$\begin{aligned} \hbox {accuracy} = \hbox {max} \frac{tr(CP)}{tr(C1_{K \times K})} \end{aligned}$$

where $P \in \{0,1\}^{K \times K}$ is the permutation matrix and $1_{K \times K}$ represents a matrix of all 1 entries.

We employed the temporal clustering algorithms on individual data streams as well as their combinations. All possible combinations from these three data streams were evaluated to find the optimum features for our task. We computed the precision and recall for the top performing set of features based on the accuracy measures.

**Table 2:** Average performance with standard deviations for various feature types tested for the different clustering algorithms using the complete dataset of nine surgeons

	SC	GMM	ACA	HACA
SSC	$71.1 \pm 16.4$	$50.6 \pm 6.2$	$70.8 \pm 17.1$	$79.0 \pm 12.3$
SI	$80.6 \pm 7.5$	$51.2 \pm 7.0$	$82.7 \pm 8.6$	$85.5 \pm 8.3$
SSC $+$ SI	$\mathbf{84.1 } \pm \mathbf{13.9 }$	$\mathbf{54.9 } \pm \mathbf{6.5 }$	$78.1 \pm 15.8$	$82.3 \pm 8.0$
SSC $+$ EVT	$72.2 \pm 14.9$	$53.6 \pm 6.0$	$73.2 \pm 13.9$	$73.9 \pm 14.6$
SI $+$ EVT	$81.9 \pm 11.2$	$53.9 \pm 6.2$	$\mathbf{82.9 } \pm \mathbf{11.0 }$	$\mathbf{88.0 } \pm \mathbf{7.1 }$
SSC $+$ SI $+$ EVT	$77.3 \pm 17.6$	$52.3 \pm 3.1$	$79.9 \pm 14.0$	$84.1 \pm 9.2$

The highest performance achieved across different features for each algorithm is shown in bold

**Table 3:** Precision and recall values for different algorithms for each task using SI $+$ EVT features

	Precision				Recall			
	SC	GMM	ACA	HACA	SC	GMM	ACA	HACA
Task1	52.4	48.8	73.2	89.2	63.1	49.3	68.3	87.4
Task2	85.0	52.7	69.3	80.3	74.6	59.5	85.7	81.5
Task3	76.6	47.5	86.4	73.7	80.3	59.7	99.7	99.7
Task4	42.1	37.8	73.0	59.7	36.0	19.6	43.3	37.3
Task5	77.9	57.4	94.8	90.0	81.2	53.6	85.8	81.1

**Fig. 4:**
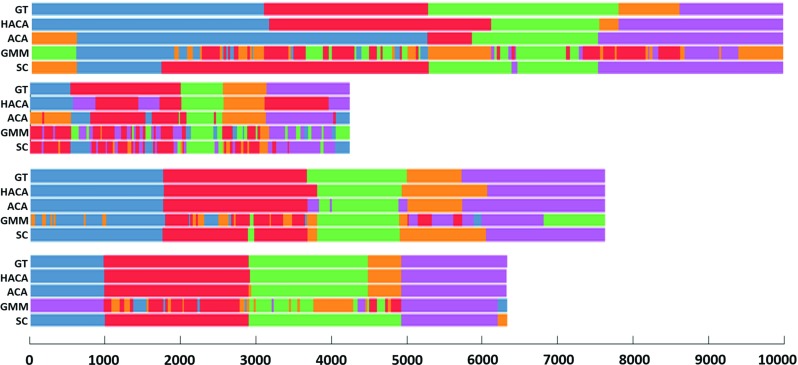
Segmentation results for four procedures. Each block contains five bars showing segmentation output using ground truth (GT), HACA, ACA, GMM, and SC

## Results and discussion

We evaluated the performance of the different unsupervised clustering algorithms described in Section “Methodology” on the surgical procedures. As described in Section “Dataset,” the dataset consisted of kinematic (pose and joint angles) and event data streams collected from the surgeon side console and the patient-side cart. We implemented the clustering algorithms on individual data streams and combinations of different data streams in order to compare how various feature sets impacted algorithm performance. Since the convergence of clustering algorithms depends on the initialization, we ran the algorithms for five different initializations and picked the solution with minimum energy (given by Eq. 3), which was the same methodology as [18]. Note that the solution that minimized the objective function also gave the highest clustering accuracy (evaluated using Eq. 6). Table 2 shows the mean accuracies achieved (over nine surgeons) for different algorithms and data streams used. Additionally, Table 3 shows the precision and recall values across tasks for the top performing data stream (SI $+$ EVT). Task4 consistently underperforms compared to the other tasks across algorithm types. Furthermore, the mean F1 score for each algorithm was: SC (0.67), GMM (0.48), ACA (0.77), and HACA (0.77). Based on these scores, ACA and HACA perform comparably but significantly outperform SC and GMM.

**Table 4:** Average performance with standard deviations for each of the five tasks (T1–T5). The feature set was SSC $+$ SI $+$ EVT

	T1	T2	T3	T4	T5
ACA	$66.4 \pm 44.3$	$71.8 \pm 33.9$	$84.8 \pm 13.6$	$74.4 \pm 45.9$	$94.6 \pm 8.0$
HACA	$87.5 \pm 35.4$	$81.2 \pm 20.4$	$80.2 \pm 20.4$	$61.7 \pm 51.0$	$88.2 \pm 26.4$

As a baseline comparison, we computed the segmentation accuracy when we simply scaled the normalized task lengths (relative to total procedure time) for each trial to estimate the transitions between tasks. The resulting accuracy is 0.60 (${\pm }0.15$) slightly better than GMM but worse than the remaining algorithms (see Table 2). This ensures the algorithms are not simply scaling tasks based on time. Although it serves a useful comparison, one can see from the example procedure bars (Fig. 4) that the duration of tasks differed for different subjects.

From the results, we can see that SC, ACA and HACA perform fairly well, while GMM performs poorly for all the feature types. As a whole, HACA outperforms all other methods for all but one feature type (SSC $+$ SI). In general, using SSC kinematic data seems to perform less well than SI, which might be because SSC contains less information than SI (i.e., hand movements versus three instrument and camera movements). Adding EVT data to SSC and SI individually improves the segmentation accuracy for most of the algorithm types but deteriorates the performance when used with the combined kinematic data (SSC $+$ SI). The highest accuracy achieved across all algorithms and features types was $88.0\%$ using HACA with SI $+$ EVT data. Our results are comparable to other surgical phase recognition methods in the literature [4, 14, 16].

Figure 4 shows example segmentation bars for four surgeons using the four different algorithms. The color scheme used for different surgical tasks in a procedure is the same as in Fig. 2. For each surgeon, the five total rows corresponded to segmentation using ground truth, HACA, ACA, GMM, and SC, respectively. One can see HACA outperforms the other methods, in general. Most misclassifications occur at the boundaries of tasks. Unlike the other methods, GMM (and to some extent SC) made many misclassifications throughout each task. In some cases, we can achieve very accurate segmentation using HACA and ACA, as shown in the lowest block in Fig. 4.

Finally, Table 4 shows the classification accuracy for each of the five tasks using ACA and HACA with the SSC $+$ SI $+$ EVT feature set. For ACA, the first tasks achieved the lowest accuracy, whereas the fifth task achieved the highest accuracy. Conversely, for HACA the fourth task achieved the lowest accuracy, whereas the fifth task achieved the highest accuracy. Across all tasks, HACA achieved a slightly more consistent classification accuracy. A one-way ANOVA showed that GMM, ACA, and HACA outperform SC across all feature types ($p<0.01$). No significant differences existed between GMM, ACA, or HACA. A two-way ANOVA for algorithm type and features showed that both the algorithm and feature type affect accuracy ($p < 0.05$) but not their interaction. Additionally, a Friedman’s test showed that algorithm type affects accuracy ($p<0.001$).

Depending on the requirements for a particular end application, some misclassification error might be tolerable around task boundaries, especially at the task-level since the duration of tasks is on the order of minutes, whereas the misclassification might be seconds. For example, compare the task boundaries between ground truth and HACA in the third surgeon in Fig. 4; the relative amount of misclassified frames is much smaller than the total width of each colored bar or task. In this way, the accuracies achieved by HACA (or ACA) could be sufficient for certain advanced analyses.

There are several limitations that exist with our analysis. Firstly, we used only five tasks to make up a procedure when most clinical procedures have more clinically discernible steps. Secondly, more formal methods could be used to optimize the parameters of the unsupervised clustering algorithms, such as a k-fold cross-validation. However, unlike supervised machine learning algorithms, the clustering algorithms used here are designed to be unsupervised and applied to situations where ground truth labels might not be available. Another limitation is that features derived from video data were not used to meet the requirement of a lightweight dataset. However, video-based features could be used to improve performance, especially when segmenting a larger number of tasks. The recent success of video-based segmentation methods also suggests it is a worthwhile endeavor [4, 14, 22]. Finally, it would be worthwhile to replicate this work on open source datasets (e.g., JIGSAWS [23]) to benchmark the performance of these algorithms against others. However, datasets such as JIGSAWS are overly simple consisting of dry-lab exercises with major limitations to system behavior (i.e., no camera movement), and therefore, algorithms applied to them can be difficult to translate to real-world environments, given the purpose of this work is to identify clinically relevant steps of procedures as opposed to low-level trajectories, such as surgemes.

Despite these limitations, our results show that RAS system data can be used by temporal clustering algorithms to accurately segment surgically realistic tasks without directly modeling low-level subtasks. We confirm that aligned clustering techniques (ACA and HACA) outperform conventional approaches like SC and GMM. Furthermore, we show that certain feature sets result in higher accuracies, and that a subset of all available features or data might be sufficient for certain applications.

## Conclusions

In this work, we examined off-line temporal clustering methods to recognize individual steps during clinically relevant training procedures in RAS. The long-term goal for this research is to provide increasingly more targeted assessment of surgical activities rather than whole procedure measures. This will enable advanced metrics to be used to benchmark and assess surgical workflow and surgeon proficiency. Our results suggest that off-line clustering methods can be used to chunk whole surgical procedures into individual, clinically relevant steps with competitive accuracies. Additionally, our approach is complementary to vision-based methods in that it uses system-based data streams present in RAS. In future studies, we plan to evaluate similar surgical phase algorithms on additional, larger datasets as well as to explore the clinical value of the step-based performance metrics on surgeon training.

## References

[1] Padoy N, Blum T, Ahmadi SA, Feussner H, Berger MO, Navab N (2012). Statistical modeling and recognition of surgical workflow. Med Image Anal.

[2] Vedula SS, Malpani A, Ahmidi N, Khudanpur S, Hager G, Chen CCG (2016). Task-level vs. segment-level quantitative metrics for surgical skill assessment. J Surg Educ.

[3] Zia A, Sharma Y, Bettadapura V, Sarin EL, Ploetz T, Clements MA, Essa I (2016). Automated video-based assessment of surgical skills for training and evaluation in medical schools. Int J Comput Assist Radiol Surg.

[4] Twinanda AP, Shehata S, Mutter D, Marescaux J, de Mathelin M, Padoy N (2016) Endonet: a deep architecture for recognition tasks on laparoscopic videos. CoRR arXiv:1602.03012 10.1109/TMI.2016.2593957 27455522

[5] Katić D, Julliard C, Wekerle AL, Kenngott H, Müller-Stich BP, Dillmann R, Speidel S, Jannin P, Gibaud B (2015). Lapontospm: an ontology for laparoscopic surgeries and its application to surgical phase recognition. Int J Comput Assist Radiol Surg.

[6] Dergachyova O, Bouget D, Huaulmé A, Morandi X, Jannin P (2016). Automatic data-driven real-time segmentation and recognition of surgical workflow. Int J Comput Assist Radiol Surg.

[7] Ahmidi N, Poddar P, Jones JD, Vedula SS, Ishii L, Hager GD, Ishii M (2015). Automated objective surgical skill assessment in the operating room from unstructured tool motion in septoplasty. Int J Comput Assist Radiol Surg.

[8] Lalys F, Bouget D, Riffaud L, Jannin P (2013). Automatic knowledge-based recognition of low-level tasks in ophthalmological procedures. Int J Comput Assist Radiol Surg.

[9] Lea C, Hager GD, Vidal R (2015) An improved model for segmentation and recognition of fine-grained activities with application to surgical training tasks. In: 2015 IEEE Winter conference on applications of computer vision, IEEE, pp 1123–1129

[10] Malpani A, Lea C, Chen CCG, Hager GD (2016). System events: readily accessible features for surgical phase detection. Int J Comput Assist Radiol Surg.

[11] Tao L, Zappella L, Hager GD, Vidal R (2013). Surgical gesture segmentation and recognition.

[12] Krishnan S, Garg A, Patil S, Lea C, Hager G, Abbeel P, Goldberg K (2015) Transition state clustering: Unsupervised surgical trajectory segmentation for robot learning. In: International symposium of robotics research. Springer STAR

[13] Krishnan S, Garg A, Patil S, Lea C, Hager G, Abbeel P, Goldberg K (2015) Unsupervised surgical task segmentation with milestone learning. In: Proceedings of international symposium on robotics research (ISRR)

[14] Despinoy F, Bouget D, Forestier G, Penet C, Zemiti N, Poignet P, Jannin P (2016). Unsupervised trajectory segmentation for surgical gesture recognition in robotic training. IEEE Trans Biomed Eng.

[15] Lea C, Reiter A, Vidal R, Hager GD (2016) Segmental spatio-temporal cnns for fine-grained action segmentation and classification. arXiv preprint arXiv:1602.02995

[16] DiPietro R, Lea C, Malpani A, Ahmidi N, Vedula SS, Lee GI, Lee MR, Hager GD (2016) Recognizing surgical activities with recurrent neural networks. In: International conference on medical image computing and computer-assisted intervention. Springer, pp 551–558

[17] Zhou F, De la Torre F, Hodgins JK (2008) Aligned cluster analysis for temporal segmentation of human motion. In: 8th IEEE international conference on automatic face & gesture recognition, 2008. FG’08., IEEE, pp 1–7

[18] Zhou F, De la Torre F, Hodgins JK (2013). Hierarchical aligned cluster analysis for temporal clustering of human motion. PAMI.

[19] Wang F, Zhang C (2005). Spectral clustering for time series.

[20] Barbič J, Safonova A, Pan JY, Faloutsos C, Hodgins JK, Pollard NS (2004) Segmenting motion capture data into distinct behaviors. In: Proceedings of Graphics Interface 2004, Canadian Human-Computer Communications Society, pp 185–194

[21] Kuhn HW (1955). The hungarian method for the assignment problem. Nav Res Logist Q.

[22] Twinanda AP, Shehata S, Mutter D, Marescaux J, de Mathelin M, Padoy N (2016) Endonet: a deep architecture for recognition tasks on laparoscopic videos. arXiv preprint arXiv:1602.03012 10.1109/TMI.2016.2593957 27455522

[23] Gao Y, Vedula SS, Reiley CE, Ahmidi N, Varadarajan B, Lin HC, Tao L, Zappella L, Béjar B, Yuh DD, Chen CCG, Vidal R, Khudanpur S, Hager GD (2014) JHU-ISI gesture and skill assessment working set (JIGSAWS): a surgical activity dataset for human motion modeling. In: MICCAI Workshop: M2CAI, vol 3

